# Attenuation and Degeneration of SARS-CoV-2 Despite Adaptive Evolution

**DOI:** 10.7759/cureus.33316

**Published:** 2023-01-03

**Authors:** Yingguang Liu

**Affiliations:** 1 Molecular and Cellular Sciences, Liberty University College of Osteopathic Medicine, Lynchburg, USA

**Keywords:** genome degradation, pneumonia, mutation, natural selection, covid-19, degeneration, attenuation, codon usage, immune evasion, evolution

## Abstract

The evolution of severe acute respiratory syndrome coronavirus 2 (SARS‑CoV‑2) has followed similar trends as other RNA viruses, such as human immunodeficiency virus type 1 and the influenza A virus. Rapid initial diversification was followed by strong competition and a rapid succession of dominant variants. Host-initiated RNA editing has been the primary mechanism for introducing mutations. A significant number of mutations detrimental to viral replication have been quickly purged. Fixed mutations are mostly diversifying mutations selected for host adaptation and immune evasion, with the latter accounting for the majority of the mutations. However, immune evasion often comes at the cost of functionality, and thus, optimal functionality is still far from being accomplished. Instead, selection for antibody-escaping variants and accumulation of near-neutral mutations have led to suboptimal codon usage and reduced replicative capacity, as demonstrated in non-respiratory cell lines. Beneficial adaptation of the virus includes reduced infectivity in lung tissues and increased tropism for the upper airway, resulting in shorter incubation periods, milder diseases, and more efficient transmission between people.

## Introduction and background

Severe acute respiratory syndrome coronavirus 2 (SARS‑CoV‑2) is one of the most intensely observed organisms in terms of evolutionary development, and coronavirus disease 2019 (COVID-19) is probably the fastest-evolving disease in terms of clinical and pathological features to be documented. Reviewing the evolution of SARS-CoV-2 not only provides insights into future trends of the pandemic and guides COVID-19 mitigation strategies but also sheds light on general features of the evolution of zoonotic RNA viruses. Based on what is currently known in the literature, this study makes the following observations concerning the direction of SARS-CoV-2 evolution. First, initial explosive diversification after entering the new host species eventually gave way to one dominant lineage, which has undergone an overall degeneration of genomic structure and loss of functionality. Second, adaptive mutations resulted in variants of lower virulence and higher transmissibility. The mechanisms underlying these trends will be discussed in this study. This article was previously posted to the Preprints server on November 23, 2022.

## Review

Degenerative evolution observed in other RNA viruses

After a zoonotic virus enters the human body, it must adapt itself to replicate in an unfamiliar host cell environment in order to evade unforeseen defense mechanisms on the cellular and organismal levels. Viruses are endowed with error-prone nucleic acid polymerases, which generate quick genetic variations among offspring. Compared to most other organisms, viruses produce higher large population surpluses to afford strong selection against low odds of survival. Even with quick mutations and large numbers of progenies, a successful jump of species is rare. Some viruses, such as avian influenza viruses, may infect humans but fail to transmit from one human body to another.

After a zoonotic virus successfully infects a human body and fortunately gains the ability to transmit between humans, it may shortly disappear from mankind. A prime example of such a virus is SARS-CoV. It appeared in humans in late 2002 and went extinct in about a year [1,2]. Other viruses may coexist with mankind for the long term and become endemic. There is evidence that zoonotic viruses that maintain high levels of transmission among humans tend to attenuate. For example, the current human coronavirus OC43 (HCov-OC43) diverged from the bovine coronavirus around 1890 [3] and probably entered mankind as the cause of the ‘Russian flu’ pandemic of 1889-1895 [4,5]. The virus has attenuated ever since and is only a cause of the common cold of the current times. Although long-term peaceful coexistence with the host is a marker of ultimate success on the part of the virus, attenuation is often accomplished by loss-of-function mutations and accompanied by compromised replication capacity, as seen in commonly used live vaccines [6-8]. 

Besides error-prone nucleic acid replication, mutations may be forced on the virus by the cellular nuclear acid-editing mechanisms. Apolipoprotein B mRNA-editing enzyme, catalytic polypeptide (APOBEC) deaminates viral cytidine to uridine. In fact, APOBEC3 is a key factor in determining the permissiveness of a cell to both DNA and RNA viruses [9,10]. Another family of nucleic acid-editing enzymes is the adenosine deaminase acting on RNA (ADAR), which, by deaminating adenosine to inosine in double-stranded RNA, affects subsequent RNA processing, translation, and stability [11]. The p150 subunit of ADAR1, as well as APOBEC3, is upregulated by type I interferons [12,13], underlining the role of nucleic acid editing in innate antiviral immunity. Although mutations introduced via nucleic acid editing can contribute to viral adaptation, directional base substitutions are more likely disruptive to genetic information. For example, biased U>C transitions of the measles virus, presumably introduced by ADAR, suppressed the translation of viral genes in infected brain tissues [14]. Interestingly, the ADAR edition of viral RNA also modulates interferon production, leading to a more peaceful coexistence between the virus and the host cell [15]. In addition, viral RNA rich in CpG dinucleotides is recognized by zinc-finger antiviral proteins [16,17]. This mechanism provides a selective advantage for CpG-depleted variants.

Whether accidentally introduced during genome replication or forced on by host-editing enzymes, mutations accumulate in viruses faster than in cellular organisms, leading to the degradation of viral genomes. During the first few months of infection by the human immunodeficiency virus (HIV), as the viral genomic sequences diversify in each patient, the overall fitness of the virus, as measured by *in vitro *viral DNA production, decreases with time [18]. During the early years of the HIV pandemic, the virus adapted its codon usage to that of human cells, but, by the end of the 20th century, the codon usage of HIV-1 showed a trend of diverging away from human codon usage patterns, indicating the degeneration of genetic information [19]. Antiretroviral drugs alter codon usage patterns [20] and result in reduced fitness of HIV-1 [21,22]. Likewise, the 1918 H1N1 influenza virus demonstrated a linear degeneration of codon scores before it finally went extinct [23].

Adaptive mutations of SARS-CoV-2 favoring upper respiratory infection

Adaptive mutations of SARS-CoV-2 are characterized by 1) convergence or homoplasy, i.e., repeated, independent emergence of the same mutations in multiple strains [24]; 2) cooccurrence of two or more mutations in fixed strains suggesting epistasis and complementarity [25]; 3) concentration of mutations in hotspots, especially in the receptor-binding domain (RBD) of the spike protein that interacts with the human angiotensin-converting enzyme 2 (ACE2) receptor and neutralizing antibodies [26]; 4) competition between variants resulting in rapid selective sweeps, which leads to new variants replacing old ones in a matter of months [27,28].

From the Lungs to the Nose

Starting with the D614G sweep early in the pandemic, almost all subsequent variants that gained dominant status harbor multiple mutations in the RBD, resulting in increases in affinity for human ACE2 by 2-4 times compared to the wild-type virus, except for the Lambda and Omicron variants where no obvious change in receptor-binding affinity was observed [29,30]. In addition, the adaption of SARS-CoV-2 includes escaping from neutralizing antibodies [31] and resisting interferons [32].

A key functional change in the spike protein of SARS-CoV-2 as it evolved is decreased tropism toward lung tissues and increased tropism toward airway epithelium. As early as 2020, it was found that the D614G mutant outcompeted the wild-type virus in primary human airway epithelial cells and the nasal epithelium, but not in the lungs, of infected hamsters [33,34]. The Beta, Delta, and Omicron variants all replicated to higher titers in ex vivo human bronchial tissues than the wild-type virus, with Omicron reaching the highest titers; however, the opposite trend was seen when the variants were tested in the lung tissues, with Omicron replicating to significantly lower titers than the wild-type virus and the Delta variant [35].

The mechanism of tropism change is still unclear. Increased binding affinity toward ACE2 favors infection of airway cells. A survey of single-cell RNA-seq data revealed that airway cells express more ACE2 than lung cells, with nasal secretory cells showing the highest expression [36]. A study using immunohistochemistry observed a similar distribution of ACE2 [37]. Local codon optimization in viral genes may also have contributed to increased replication efficiency, especially at key genomic sites, for instance, the hexanucleotides encoding the arginine dimer of the furin-cleavage site of the spike protein [38]. Reduced replicative capacity in the alveolar epithelium may be related to a documented change in viral entry. SARS-CoV-2 uses the transmembrane serine protease 2 (TMPRSS2) to cleave the S2 site of its spike protein to yield an S2 subunit that can induce fusion between the viral envelope and the cell membrane [39]. It has been demonstrated that the Omicron variant has lost its sensitivity to TMPRSS2 and, consequently, lost its ability to induce membrane fusion [40]. The variant is now more dependent on passive endocytosis as its mode of entry. This is demonstrated by increased sensitivity to inhibitors of endosomal proteases such as chloroquine [41]. Loss of a primary means of cell entry conceivably compromises viral infectivity, especially in alveolar cells where the concentration of ACE2 is low. In addition, alveolar and airway epithelial cells may have different capacities in carrying out receptor-mediated endocytosis, and the local defense mechanisms of the lungs may be more efficient in resisting the mutated viruses.

This change in tissue tropism is reflected in the clinical features of COVID-19. Infection with the Omicron variant is characterized by upper respiratory symptoms such as rhinorrhea, sore throat, sneezing, and hoarse voice, while the absence of anosmia and ageusia may reflect reduced tropism toward extra-respiratory tissues [42]. Moreover, reportedly, the Omicron variant is less likely to cause severe pneumonia than the Delta variant even after adjustment for vaccination status in multivariable analysis [43]. In patients infected with the Omicron variant, pneumonia was more strongly associated with patients of old age, male gender, and with diabetes than those infected with previous variants [44]. The Alpha and Beta variants, but not the Delta variant, were also less likely to cause pneumonia than the wild-type virus even after adjustment for age, sex, comorbidities, and vaccination status [45]. Morbidity and mortality of COVID-19 are associated more with immune dysregulation than with viral replication, but excessive activation of immune cells typically follows viral infection of the lungs and the subsequent accumulation of macrophages and neutrophils in the lungs [46,47].

Increasing Transmissibility

Since there are only a few ACE2 receptors in the lungs, even SARS-CoV-2 strains that are not variants of concern exhibit lower titers in the lungs than in the airway, as seen in hamsters and organoid cultures in previous studies [31,48]. Therefore, increased tropism toward the upper respiratory tract likely results in higher viral productivity. Additionally, it also means quicker access to permissive cells and easier viral shedding, which, in turn, means shortened incubation periods and higher transmissibility. The incubation period of the wild-type virus among travelers from Wuhan, China, ranged from 2.1 to 11.1 days, with a mean of 6.4 days [49]. One meta-analysis of the incubation period caused by the later variants found the mean incubation period of the Alpha, Beta, Delta, and Omicron variants to be 5.00 days, 4.50 days, 4.41 days, and 3.42 days, respectively [50]. In comparison, the median incubation period of the human coronaviruses that cause the common cold is only 3.2 days [51].

Sungnak et al. [32] compared the transmissibility of several respiratory viruses whose receptors are distributed differently within the respiratory system and found that tropism toward the upper respiratory tract is associated with higher transmissibility. Loss of replicative function in the lungs results in milder diseases, allowing the patients to be more ambulatory and, thus, spread the virus to a greater extent. Indeed, later variants of SARS-CoV-2 tend to show increased transmissibility. Using two mathematical models to calculate the effective reproduction number (Rt), Hasan et al. [52] demonstrated the increased transmissibility of SARS-CoV-2 in Scandinavia as the Delta variant replaced earlier variants in 2021. The basic reproduction number (R0) of the wild-type virus ranged from 0.47 to 6.47, with an average of 2.69 [53]. Using a competition model, Hansen et al. [54] calculated the relative transmissibility of the Alpha, Delta, and Omicron variants compared with the wild-type virus and found that the Rt of the three variants were 1.51, 3.28, and 10.33 times that of the wild-type virus, respectively.

Enhanced transmissibility is obviously the most important factor in terms of selective advantage against other variants. Theoretically, there is an optimum configuration of the spike protein that interacts with the human ACE2 and proteases with maximum efficiency, and there is an optimum mode of entry that results in maximum tropism toward the nasal epithelium and minimum tropism toward the alveolar epithelium. By *in vitro* evolution and testing, Zahradník et al. [55] produced an ideal RBD model called RBD-62 that has an ACE2-binding affinity that is 1000-fold higher than that of the wild-type virus. RBD-62 contains a combination of S477N, Q498R, and N501Y mutations, which were all found later in the Omicron variant [56]. In fact, most mutations that could increase the binding affinity between the RBD and ACE2 had already emerged before Omicron [57]. The R0 of the Omicron variant is already at par with the most transmissible respiratory virus known to date, that is, the measles virus [58]. Can the transmissibility be further improved?

Degenerative evolution of SARS-CoV-2

The virus still has room to improve, but nature may not allow that to happen. Although some gain-of-function mutations have been realized, they tend to be outnumbered by functionally destructive mutations.

The most dominant mechanism that drives mutations in the genome of SARS-CoV-2 is RNA editing. The most common mutation seen in the latter variants is the C>U transition, which is presumably induced by the APOBEC enzymes [23,59]. When APOBEC deaminates cytosine in the antigenome (the replication intermediate, or the negative strand), it results in the G>A transition in the viral genome. ADAR induces the A>G transition when it deaminates adenine in the genome and induces the U>C transition for the same in the antigenome. Giorgio et al. [60] studied the editing of SARS-CoV-2 RNA in the transcriptome of bronchoalveolar lavage samples of infected patients and found high levels of A>G and U>C transitions that are indicative of ADAR action. The numbers of A>G and U>C transitions are roughly equal, consistent with the fact that ADAR acts on dsRNA. They also found significant C>U transitions, which are more in number than G>A transitions, indicating APOBEC editing and its preference for the positive strand. In comparison, substitution patterns characteristic of replication errors introduced by the viral RNA-dependent RNA polymerase (C>A, U>C, G>U, A>C, and U>G) are much rarer in the transcriptome. Kim et al. [61] also demonstrated APOBEC enzymes and SARS-CoV-2 RNA segments in HEK293T cells and proved that APOBEC3A, APOBEC1, and APOBEC3G can all edit SARS-CoV-2 RNA. One signature mutation caused by APOBEC3A in the 5’ untranslated region appeared early in 2020 and has been fixed in dominant variants, including the Delta and Omicron variants. One signature mutation pattern of APOBEC1 contributed to the H655Y mutation in the spike protein, which is associated with the loss of TMPRSS2 usage. APOBEC3A also significantly increased viral yield in Caco-2 cells infected with SARS-CoV-2. These findings indicate that the virus can take advantage of the host cell RNA editing enzymes for adaptive evolution. However, as expected, most mutations turn out to be destructive and subject to purifying selection [62-64], whether they are imposed on the virus in the host or due to errors during viral replication.

Darwinian Mechanisms That Drive Degeneration of Genetic Information

**Figure 1:**
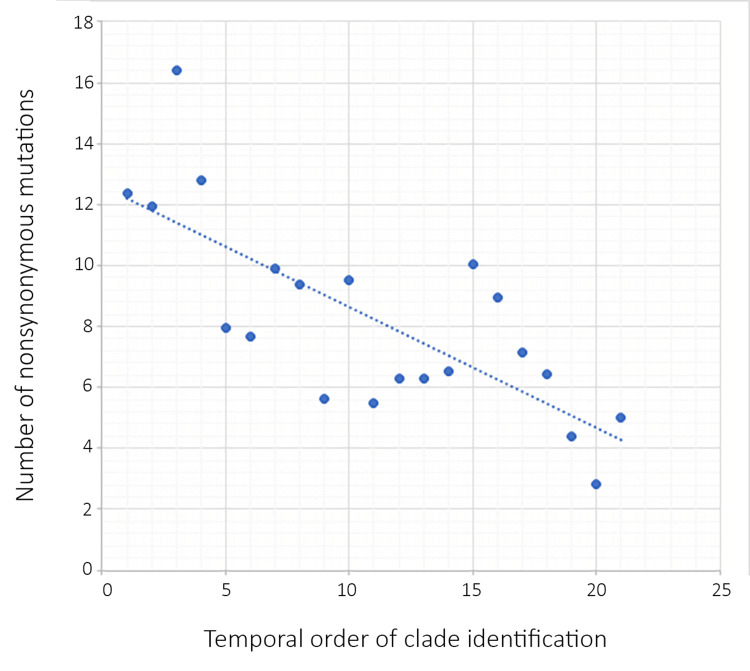
Decline of intra-clade mutation rate from early to later clades.

Natural selection for immune evasion impedes the functionality of genes: SARS-CoV-2 has accumulated far more nonsynonymous mutations than synonymous mutations, indicating positive selection [65-67]. Amino acid substitutions presumably led to rapid diversification early in the pandemic as a result of the virus adapting to the new host species, and immune evasion has been a driving force since 2020 [68]. Most mutations concentrate on the spike protein, which is the main target of antibodies. The Omicron variant (BA.1) has 15 mutations in the RBD, far more than the one to four mutations occurring in any other major variants. Six of the 15 mutations are known to increase affinity toward ACE2, while nine are known to decrease this affinity [56]. The overall affinity of the spike protein of the Omicron variant, as measured experimentally, is at par with that of the wild-type virus [56,69]. The effects of destructive mutations are balanced by constructive mutations, but the destructive mutations allow for the evasion of pre-existing neutralizing antibodies in the population [56]. The idea that most mutations in the RBD decrease affinity to ACE2 has been corroborated by Li et al. in the early part of the pandemic. Using vesicular stomatitis virus pseudotyped with the spike protein of SARS-CoV-2, the research group measured the infectivity of 51 variants with mutations in the RBD and found 13 mutations resulting in decreased infectivity while only three led to increased infectivity. Some mutations that decreased infectivity enabled the spike protein to evade antibodies. The majority of the RBD mutations did not yield a measurable phenotypic difference in binding affinities [70]. Theoretically, there is a limited number of epitopes that can be altered without seriously impeding viral replication. Therefore, the rate at which the virus accumulates immunity-escaping mutations must slow down with time. Neher [63] compared the synonymous and nonsynonymous mutation rates of the viral clades that arose between 2019 and 2022. Synonymous mutation rates remained constant at about five to eight base changes per genome per year for all clades, but nonsynonymous mutation rates dropped from the highest of 16.41 per genome per year (clade 19B++) to the lowest of 2.81 per genome per year (clade 22A). Figure 1 has been plotted using Neher’s data, demonstrating a negative correlation between intraclade mutation rates and time.

Consistent with the fact that there is a slower accumulation of additional mutations in later clades, we observe the fixation of a reverse mutation (R493Q) in the spike protein of the Omicron subvariant, BA.2.75, although the reversion reduced resistance to neutralizing antibodies [71], indicating the possibility of cyclic evolution.

Therefore, even if the ideal viral genome ever appears through mutation and recombination, it will soon degenerate because the virus needs to continue mutating to evade a practically infinite inventory of antibodies and antiviral T lymphocytes in the human population.

Accumulation of non-selectable mutations: Although nonsynonymous mutations concentrate on the spike protein, they accumulate in other genes throughout the viral genome as well. Unlike the surface proteins and the RNA polymerase, accessory proteins demonstrate little restraint on amino acid substitutions [63,72]. Accumulation of mutations in proteins that are loosely selected may contribute to genetic degradation in the long term. Synonymous mutations affect codon usage adaptation and translation efficiency [73,74]. While synonymous mutations that promote adaptation to human codon usage show a tendency to increase, most synonymous mutations, mainly those induced by APOBEC enzymes, are oblivious to natural selection [73]. Since C>U transitions are significantly the most abundant base substitutions in the mutational spectrum of SARS-CoV-2 [75-77], uracil steadily accumulates in the viral genome, while the number of cytosine declines with time [78]. Since intramolecular base-pairing forms secondary structures, which can stabilize the genome, and AU bonding is less tight than GC bonding, C>U transitions also destabilize the viral genome, which may facilitate nucleotide deletions, leading to slightly shorter genomes in all major variants (Table 1) [78].

**Table 1:** Nucleotide deletions in major variants of concern compared to early clade consensus.

	Alpha	Beta	Gamma	Delta	Omicron
Consensus genome length	29,750	29,751	29,764	29,756	29,742
Deletions	19	18	5	13	27

SARS-CoV-2 still has a higher cytosine count and lower uracil count than any of the four human coronaviruses [79], which leads us to the following question: Will SARS-CoV-2 continue to evolve toward the nucleotide compositions of the current human coronaviruses and become more like them phenotypically?

Phenotypic Loss Due to Genetic Degradation

Codon degeneration and reduced translation efficiency: Both synonymous and nonsynonymous mutations may deoptimize codon usage. In 2020, early in the pandemic, Posani et al. [80] found a general trend of codon deoptimization among most genes of SARS-CoV-2, and the effective number of codons (ENC) plot was indicated natural selection as a driving force of codon deoptimization, especially for genes of the spike protein and the RNA-dependent RNA polymerase [80]. Mogro et al. [81] discovered a downward trend of codon adaptation index (CAI) of SARS-CoV-2 in multiple human tissues, and surprisingly, the Omicron variant (BA.1) had a higher CAI than other variants of concern, although lower than the earliest isolates. Wu et al. [82] experimentally demonstrated higher protein expression when codon usage was restored to optimum and found a sudden increase in CAI with the emergence of the Omicron variant. The high CAI of Omicron may shed light on the mysterious origin of the variant [83]. If the variant was generated in a chronically infected, immunodeficient person, the nucleic acid-editing enzymes may still have caused codon degeneration unless the deficiency had to do with RNA editing. On the other hand, we know that bat coronaviruses have higher CpG content, a higher percentage of C, and a lower percentage of U [84]. The high CAI of Omicron favors the “reverse zoonosis” hypothesis. Other studies have proposed that the variant was a recombinant between an ancestral lineage and a hypermutated virus [72], but how the ancestral lineage was spared from codon degeneration is still unknown. Nonetheless, among humans, the future of the Omicron variant is most likely codon deoptimization and further attenuation.

**Figure 2:**
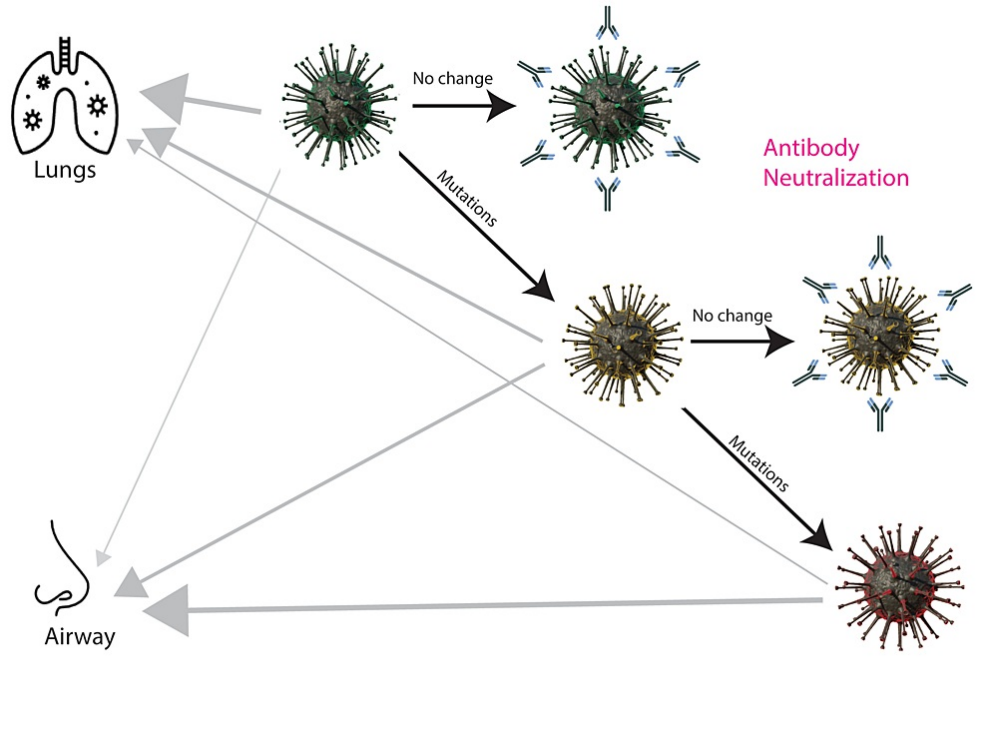
Degenerative mutations of SARS-CoV-2, the role of selection by immune evasion, and the change in tissue tropism. Dark arrows indicate viral evolution, while gray arrows indicate tissue tropism. The thickness of the gray arrows represents the relative intensity of tropism. Phenotypic consequences of mutations are represented by the colors of the viral particles. Neutralizing antibodies are drawn around unchanged viruses. The figure is the author’s own creation with the assistance of scientific illustrator Peng Wang.

Reduced replication capacity in non-respiratory cell lines: Through adaptation, newer SARS-CoV-2 variants have lost replication efficiency in lung cells and gained efficiency to replicate in airway cells. Comparison of the overall genomic functionality of early and late variants should, therefore, be conducted in cells that none of them has been exposed to or in cell lines where the variants have replicated for similar periods of time, for example, the monkey kidney cell line, Vero. Using Vero E6 cells, Mautner et al. [85] showed that the replication of the B.1.1 strain (not a variant of concern) was the fastest, while that of Omicron was the slowest [85]. The difference between the Delta variant and the Omicron variant was greater when they were compared in Vero E6 cells expressing TMPRSS2 and it was found that Omicron lost its ability to use TMPRSS2 for cell entry [40]. Omicron also showed a severely weakened ability to cause cytopathic effects, including cell fusion, in Vero cells. Caco-2 is a human colon epithelial carcinoma cell line. When the infectivity of SARS-CoV-2 variants was compared in Caco-2, Omicron replicated at a drastically slower pace than any other variant and produced very low titers of progeny virus in the end [85]. This may be a clue to explain the narrower tissue specificity of Omicron in comparison with earlier variants. The pathology of Omicron infection is more localized to the respiratory system, resulting in fewer symptoms in the digestive and nervous systems [42,86,87]. Interestingly, in the airway epithelial carcinoma cell line Calu-3, the deficiency of Omicron was less dramatic, presumably due to its adaptation to airway cells [85]. Figure 2 summarizes the degenerative evolution of SARS-CoV-2 under immune pressure.

## Conclusions

Like other zoonotic RNA viruses, SARS-CoV-2 is evolving toward attenuation and genetic degradation. The most important cause of mutations is host-initiated RNA editing, and the most important force driving mutation accumulation is immune evasion. In fact, immune evasion and accumulation of near-neutral mutations both compromise the functionality of viral genes, leading to codon deoptimization and reduced replication capacity. Meanwhile, loss of sensitivity to the TMPRSS2 protease and increased affinity to the ACE2 receptor probably contributed to the change in tissue tropism, resulting in more symptoms of the upper respiratory tract, fewer cases of pneumonia, less involvement of other systems, shorter incubation periods, and enhanced transmissibility. 

Intensive observation of SARS-CoV-2 during COVID-19 may shed light on the evolution of other zoonotic RNA viruses of similar virulence and similar mode of transmission, but caution must be exercised when attempting to extrapolate these observations, especially in the case of non-zoonotic viruses or DNA viruses.

Relatively rapid attenuation of SARS-CoV-2 in the general population also implies more dangerous consequences of potential laboratory infections by earlier, more virulent variants. As herd immunity drifts toward resisting later variants, laboratory leaks of earlier variants may initiate new waves of epidemics caused by more virulent strains. Strict laboratory regulations are required to be implemented since lab-acquired infections of SARS-CoV happened multiple times in 2003 and 2004.
